# Segment-Tube: Spatio-Temporal Action Localization in Untrimmed Videos with Per-Frame Segmentation

**DOI:** 10.3390/s18051657

**Published:** 2018-05-22

**Authors:** Le Wang, Xuhuan Duan, Qilin Zhang, Zhenxing Niu, Gang Hua, Nanning Zheng

**Affiliations:** 1 Institute of Artificial Intelligence and Robotics, Xi’an Jiaotong University, Xi’an, Shannxi 710049, China; duanxuhuan0123@stu.xjtu.edu.cn (X.D.); nnzheng@xjtu.edu.cn (N.Z.); 2 HERE Technologies, Chicago, IL 60606, USA; qilin.zhang@here.com; 3 Alibaba Group, Hangzhou 311121, China; zhenxing.nzx@alibaba-inc.com; 4 Microsoft Research, Redmond, WA 98052, USA; ganghua@microsoft.com

**Keywords:** action localization, action segmentation, 3D ConvNets, LSTM

## Abstract

Inspired by the recent spatio-temporal action localization efforts with tubelets (sequences of bounding boxes), we present a new spatio-temporal action localization detector Segment-tube, which consists of sequences of per-frame segmentation masks. The proposed Segment-tube detector can temporally pinpoint the starting/ending frame of each action category in the presence of preceding/subsequent interference actions in untrimmed videos. Simultaneously, the Segment-tube detector produces per-frame segmentation masks instead of bounding boxes, offering superior spatial accuracy to tubelets. This is achieved by alternating iterative optimization between temporal action localization and spatial action segmentation. Experimental results on three datasets validated the efficacy of the proposed method, including (1) temporal action localization on the THUMOS 2014 dataset; (2) spatial action segmentation on the Segtrack dataset; and (3) joint spatio-temporal action localization on the newly proposed ActSeg dataset. It is shown that our method compares favorably with existing state-of-the-art methods.

## 1. Introduction

Joint spatio-temporal action localization has attracted significant attention in recent years [1,2,3,4,5,6,7,8,9,10,11,12,13,14,15,16,17,18], whose objectives include action classification (determining whether a specific action is present), temporal localization (pinpointing the starting/ending frame of the specific action) and spatio-temporal localization (typically bounding box regression on 2D frames, e.g., [6,12]). Such efforts include local feature based methods [1], convolution neural networks (ConvNets or CNNs) based methods [2,14,15], 3D ConvNets based methods [4,11] and its variants [19,20,21]. Recently, long short-term memory (LSTM) based recurrent neural networks (RNNs) are added on top of CNNs for action classification [5] and action localization [7].

Despite the successes of the prior methods, there are still several limiting factors impeding practical applications. On the one hand, a large number of methods [2,3,5,13] conduct action recognition only on trimmed videos, where each video contains only one action without interferences from other potentially confusing actions. On the other hand, many methods [1,7,8,9,10,11,15,16,17] emphasize only on temporal action localization with untrimmed videos, without depicting the spatial locations of the target action in each video frame.

Although there are several tubelet-style (which outputs sequences of bounding boxes) spatio-temporal action localization efforts [6,12,22], they are restricted to trimmed video only. For practical applications, untrimmed videos are much more prevalent, and sequences of bounding boxes might not offer enough spatial accuracy, especially for irregular shapes. This motivated us to propose a practical spatio-temporal action localization method, which is capable of spatially and temporally localizing the target actions with per-frame segmentation in untrimmed videos.

With applications in untrimmed videos with improved spatial accuracy in mind, we propose the spatio-temporal action localization detector Segment-tube, which localizes target actions as sequences of per-frame segmentation masks instead of sequences of bounding boxes.

**Figure 1:**
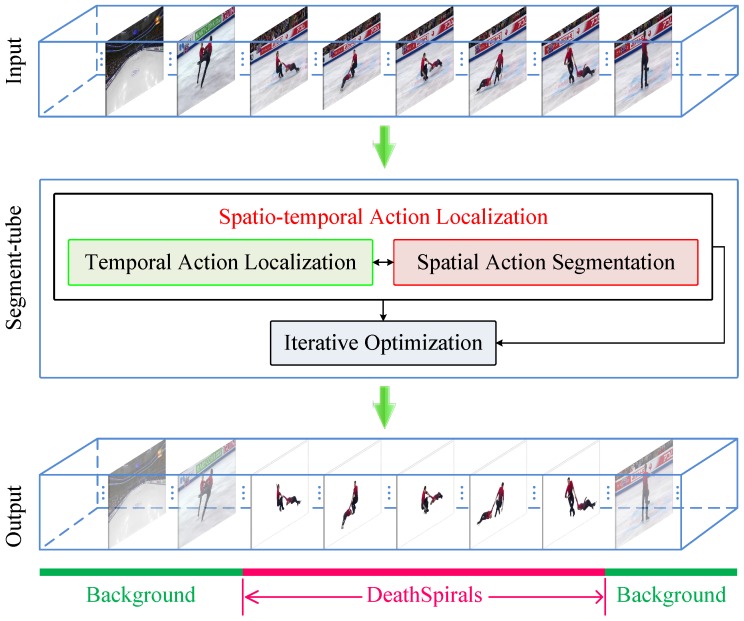
Flowchart of the proposed spatio-temporal action localization detector Segment-tube. As the input, an untrimmed video contains multiple frames of actions (e.g., all actions in a pair figure skating video), with only a portion of these frames belonging to a relevant category (e.g., the DeathSpirals). There are usually irrelevant preceding and subsequent actions (background). The Segment-tube detector alternates the optimization of temporal localization and spatial segmentation iteratively. The final output is a sequence of per-frame segmentation masks with precise starting/ending frames denoted with the red chunk at the bottom, while the background are marked with green chunks at the bottom.

The proposed Segment-tube detector is illustrated in Figure 1. The sample input is an untrimmed video containing all frames in a pair figure skating video, with only a portion of these frames belonging to a relevant category (e.g., the DeathSpirals). Initialized with saliency [23] based image segmentation on individual frames, our method first performs temporal action localization step with a cascaded 3D ConvNets [4] and LSTM, and pinpoints the starting frame and the ending frame of a target action with a coarse-to-fine strategy. Subsequently, the Segment-tube detector refines per-frame spatial segmentation with graph cut [24] by focusing on relevant frames identified by the temporal action localization step. The optimization alternates between the temporal action localization and spatial action segmentation in an iterative manner. Upon practical convergence, the final spatio-temporal action localization results are obtained in the format of a sequence of per-frame segmentation masks (bottom row in Figure 1) with precise starting/ending frames. Intuitively, the temporal action localization and spatial action segmentation naturally benefit each other.

We conduct experimental evaluations (in both qualitative and quantitative measures) of the proposed Segment-tube detector and existing state-of-the-art methods on three benchmark datasets, including (1) temporal action localization on the THUMOS 2014 dataset [25]; (2) spatial action segmentation on the SegTrack dataset [26,27]; and (3) joint spatio-temporal action localization on the newly proposed ActSeg dataset, which is a newly proposed spatio-temporal action localization dataset with per-frame ground truth segmentation masks, and it will be released on our project website. The experimental results show the performance advantage of the proposed Segment-tube detector and validate its efficacy in spatio-temporal action localization with per-frame segmentation.

In summary, the contributions of this paper are as follows:

- The spatio-temporal action localization detector Segment-tube is proposed for untrimmed videos, which produces not only the starting/ending frame of an action, but also per-frame segmentation masks instead of sequences of bounding boxes.
- The proposed Segment-tube detector achieves collaborative optimization of temporal localization and spatial segmentation with a new iterative alternation approach, where the temporal localization is achieved by a coarse-to-fine strategy based on cascaded 3D ConvNets [4] and LSTM.
- To exactly evaluate the proposed Segment-tube and to build a benchmark for future research, a new ActSeg dataset is proposed, which consists 641 videos with temporal annotations and per-frame ground truth segmentation masks.

The remainder of the paper is organized as follows. In Section 2, we review the related work. In Section 3, we present the problem formulation for spatio-temporal action localization with per-frame segmentation. In Section 4, the experimental results are presented with additional discussions. Finally, the paper is concluded in Section 5.

## 2. Related Works

The joint spatio-temporal action localization problem involves three distinctive tasks simultaneously, i.e., action classification, temporal action localization, and spatio-temporal action localization. Brief reviews of related works on these three topics are first provided. In addition, relevant works in video object segmentation are also introduced in this section.

### 2.1. Action Classification

The objective of action classification is to determine the presence of a specific action (e.g., jump and pole vault) in a video. A considerable amount of previous efforts are limited to action classification in manually trimmed short videos [2,3,5,13,28,29], where each video clip contains one and only one action, without possible interferences from either proceeding/subsequent actions or complex background.

Many methods [1] rely on handcrafted local invariant features, such as histograms of image gradients (HOG) [30], histograms of flow (HOF) [31] and improved Dense Trajectory (iDT) [28]. Video representations are typically built on top of these features by the Fisher Vector (FV) [32] or Vector of Linearly Aggregated Descriptors (VLAD) [33] to determine action categories. Recently, CNNs based methods [2,14,15] have enabled the replacement of handcrafted features with learned features, and they have achieved impressive classification performance. 3D ConvNets based methods [4,19,20,21] are also proposed to construct spatio-temporal features. Tran et al. [4] demonstrated that 3D ConvNets are good feature learning machines that model appearance and motion simultaneously. Carreira et al. [19] proposed a new two-stream Inflated 3D ConvNet (I3D) architecture for action classification. Hara et al. [21] discovered that 3D architectures (two-stream I3D/ResNet/ResNeXt) pre-trained on Kinetics dataset outperform complex 2D architectures. Subsequently, long short-term memory (LSTM)-based recurrent neural networks (RNNs) are added on top of CNNs to incorporate longer term temporal information and better classify video sequences [5,7].

### 2.2. Temporal Action Localization

Temporal action localization aims at pinpointing the starting and ending frames of a specific action in a video. Much progress has been made recently, thanks to plenty of large-scale datasets including the THUMOS dataset [25], the ActivityNet dataset [34], and the MEXaction2 dataset [35]. Most state-of-the-art methods are based on sliding windows [1,11,32,36,37], frame-wise predictions [7,15,38,39], or action proposals [22,40,41].

The sliding window-based methods typically exploit fixed-length temporally sliding windows to sample each video. They can leverage the temporal dependencies among video frames, but they commonly lead to higher computational cost due to redundancies in overlapping windows. Gaidon et al. [36] used sliding window classifiers to locate action parts (actoms) from a sequence of histograms of actom-anchored visual features. Oneata et al. [32] and Yuan et al. [37] both used sliding window classifiers on FV representations of iDT features. Shou et al. [11] proposed a sliding window-style 3D ConvNet for action localization without relying on hand-crafted features or FV/VLAD representations.

The frame-wise predictions-based methods classifies each individual video frame (i.e., predicts whether a specific category of action is present), and aggregate such predictions temporally. Singh et al. [38] used a frame-wise classifier for action location proposal, followed by a temporal aggregation step that promotes piecewise smoothness in such proposals. Yuan et al. [15] proposed to characterize the temporal evolution as a structural maximal sum of frame-wise classification scores. To account for the dynamics among video frames, RNNs with LSTM are typically employed. In [7,39], an LSTM produced detection scores of activities and non-activities based on CNN features at every frame. Although such RNNs can exploit temporal state transitions over frames for frame-wise predictions, their inputs are frame-level CNN features computed independently on each frame. On contrary in this paper, we leverage 3D ConvNets with LSTM to capture the spatio-temporal information from adjacent frames.

The action proposals-based methods leverage temporal action proposals instead of video clips for efficient action localization. Jain et al. [22] produced tubelets ( i.e., 2D + t sequences of bounding boxes) by merging a hierarchy of super-voxels. Yu and Yuan [40] proposed the actionness score and a greedy search strategy to generate action proposals. Buch et al. [41] introduced a temporal action proposals generation framework that only needs to process the entire video in a single pass.

### 2.3. Spatio-Temporal Action Localization

There are many publications about the spatio-temporal action localization problem [6,12,42,43,44,45]. Soomro et al. [43] proposed a method based on super-voxel. Several methods [6,44] formulated spatio-temporal action localization as a tracking problem with object proposal detection at each video frame and sequences of bounding boxes as outputs. Kalogeiton et al. [12] proposed an action tubelet detector that takes a sequence of frames as input and produces sequences of bounding boxes with improved action scores as outputs. Singh et al. [45] presented an online learning framework for spatio-temporal action localization and prediction. Despite their successes, all the aforementioned spatio-temporal action localization methods require trimmed videos as inputs, and only output tubelet-style boundaries of an action, i.e., sequences of bounding boxes.

In contrast, we propose the spatio-temporal action localization detector Segment-tube for untrimmed videos, which can provide per-frame segmentation masks instead of sequences of bounding boxes. Moreover, to facilitate the training of the proposed Segment-tube detector and to establish a benchmark for future research, we introduce a new untrimmed video dataset for action localization and segmentation (i.e., ActSeg dataset), with temporal annotations and per-frame ground truth segmentation masks.

### 2.4. Video Object Segmentation

Video object segmentation aims at separating the object of interest from the background throughout all video frames. Previous video object segmentation methods can be roughly categorized into the unsupervised methods and the supervised counterparts.

Without requiring labels/annotations, unsupervised video object segmentation methods typically exploit features such as long-range point trajectories [46], motion characteristics [47], appearance [48,49], or saliency [50]. Recently, Jain et al. [51] proposed an end-to-end learning framework which combines motion and appearance information to produce a pixel-wise binary segmentation mask for each frame.

Differently, supervised video object segmentation methods do require user annotations of a primary object ( i.e., the foreground), and the prevailing methods are based on label propagation [52,53]. For example, Marki et al. [52] utilize the segmentation mask of the first frame to construct appearance models, and the inference for subsequent frames are obtained by optimizing an energy function on a regularly sampled bilateral grid. Caelles et al. [54] adopted the Fully Convolutional Networks (FCNs) to tackle video object segmentation, given the segmentation mask for the first frame.

However, all the above video object segmentation methods assume that the object of interest (or primary object) consistently appears throughout all video frames, which is reasonable for manually trimmed video dataset. On the contrary, for practical applications with user-generated, noisy untrimmed videos, this assumption seldom holds true. Fortunately, the proposed Segment-tube detector eliminates such a strong assumption, and it is robust to irrelevant video frames and can be utilized to process untrimmed videos.

## 3. Problem Formulation

Given a video $V={\left\{f_{t}\right\}}_{t=1}^{T}$ consisting of *T* frames, our objective is to determine whether a specific action $k\in \left\{1,\ldots ,K\right\}$ appears in *V*, and if so, temporally pinpoint the starting frame $f_{s}\left(k\right)$ and ending frame $f_{e}\left(k\right)$ for action *k*. Simultaneously, a sequence of segmentation masks $B={\left\{b_{t}\right\}}_{t=f_{s}\left(k\right)}^{f_{e}\left(k\right)}$ within such frame range should be obtained, with $b_{t}$ being a binary segmentation label for frame $f_{t}$. Practically, $b_{t}$ consists of a series of superpixels $b_{t}={\left\{b_{t,i}\right\}}_{i=1}^{N_{t}}$, with $N_{t}$ being the total number of superpixels in frame $f_{t}$.

### 3.1. Temporal Action Localization

**Figure 2:**
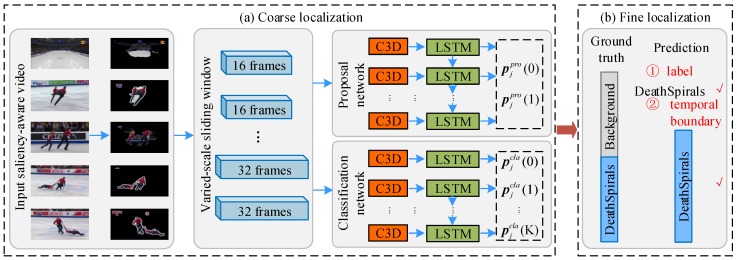
Overview of the proposed coarse-to-fine temporal action localization. (**a**) coarse localization. Given an untrimmed video, we first generate saliency-aware video clips via variable-length sliding windows. The proposal network decides whether a video clip contains any actions (so the clip is added to the candidate set) or pure background (so the clip is directly discarded). The subsequent classification network predicts the specific action class for each candidate clip and outputs the classification scores and action labels. (**b**) fine localization. With the classification scores and action labels from prior coarse localization, further prediction of the video category is carried out and its starting and ending frames are obtained.

A coarse-to-fine action localization strategy is implemented to accurately find the temporal boundaries of the target action *k* from an untrimmed video, as illustrated in Figure 2. This is achieved by a cascaded 3D ConvNets with LSTM. The 3D ConvNets [4] consists of eight 3D convolution layers, five 3D pooling layers, and two fully connected layers. The fully-connected 7th layer activation feature is used to represent the video clip. To exploit the temporal correlations, we incorporate a two-layer LSTM [5] using the Peephole implementation (with 256 hidden states in each layer) with 3D ConvNets.

**Coarse Action Localization**. The coarse action localization determines the approximate temporal boundaries with a fixed step-size ( i.e., video clip length). We first generate a set of *U* saliency-aware video clips ${\left\{u_{j}\right\}}_{j=1}^{U}$ with variable-length (e.g., 16 and 32 frames per video clip) sliding window with 75% overlap ratio on the initial segmentation $B_{o}$ of video *V* (by using saliency [23]), and proceed to train a cascaded 3D ConvNets with LSTM that couples a proposal network and a classification network.

The proposal network is action class-agnostic, and it determines whether any actions ($\forall k\in \left\{1,\ldots ,K\right\}$) are present in video clip $u_{j}$. The classification network determines whether a specific action *k* is present in video clip $u_{j}$. We follow [11] to construct the training data from these video clips. The training details of the proposal network and classification network are presented immediately below in Section 4.2.

Specifically, we train the proposal network (a 3D ConvNets with LSTM) to score each video clip $u_{j}$ with a proposal score $p_{j}^{pro}={\left[p_{j}^{pro}\left(1\right),p_{j}^{pro}\left(0\right)\right]}^{T}\in R^{2}$. Subsequently, a flag label $l_{j}^{fla}$ is obtained for each video clip $u_{j}$,

(1) $$l_{j}^{fla}=\left\{\begin{array}{cc} 1, & \;\mathrm{if}\;p_{j}^{pro}\left(1\right)>p_{j}^{pro}\left(0\right),\\ 0, & \;\mathrm{otherwise}, \end{array}\right.$$

where $l_{j}^{fla}=1$ denotes the video clip $u_{j}$ contains an action ($\forall k\in \left\{1,\ldots ,K\right\}$), and $l_{j}^{fla}=0$ otherwise.

A classification network (also a 3D ConvNets with LSTM) is further trained to predict a (K+1)- dimensional classification score $p_{j}^{cla}$ for each clip that contains an action $\left\{u_{j}|l_{j}^{fla}=1\right\}$, based on which a specific action label $l_{j}^{spe}\in {\left\{k\right\}}_{k=0}^{K}$ and score $v_{j}^{spe}\in \left[0,1\right]$ for $u_{j}$ are assigned,

(2) $$\begin{array}{ccc} l_{j}^{spe} & = & \arg\max_{k=0,\ldots ,K}p_{j}^{cla}\left(k\right), \end{array}$$

(3) $$\begin{array}{ccc} v_{j}^{spe} & = & \max_{k=0,\ldots ,K}p_{j}^{cla}\left(k\right). \end{array}$$

where category 0 denotes the additional “background” category. Although the proposal network prefilters most “background” clips, a background category is still needed for robustness in the classification network.

**Figure 3:**
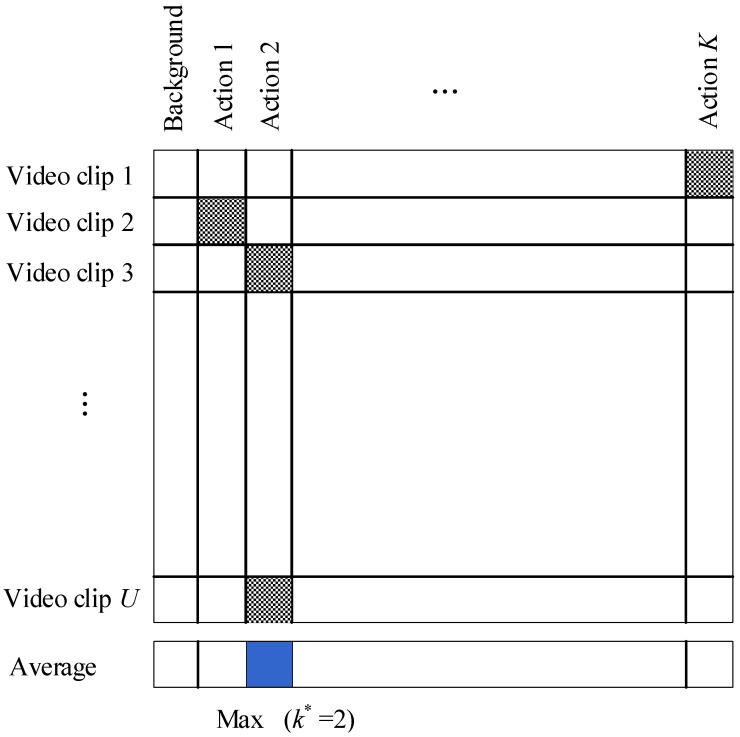
The diagrammatic sketch on the determination of video category $k^{*}$ from video clips.

**Fine Action Localization**. With the obtained per-clip specific action labels $l_{j}^{spe}$ and $v_{j}^{spe}$, the fine action localization step predicts the video category $k^{*}$ ($k^{*}\in \left\{1,\ldots ,K\right\}$), and subsequently obtains its starting frame $f_{s}\left(k^{*}\right)$ and its ending frame $f_{e}\left(k^{*}\right)$. We calculate the average of specific action scores $v_{j}^{spe}$ over all video clips for each specific action label $l_{j}^{spe}$, and take the label $k^{*}$ with the maximum average predicted score as the predicted action, as illustrated in Figure 3.

Subsequently, we average specific action scores $v_{j}^{spe}$ of each frame $f_{t}$ for the label $k^{*}$ in different video clips to obtain the action score $\alpha_{t}\left(f_{t}\right)$ for frame $f_{t}$. By selecting an appropriate threshold we can obtain the action label $l_{t}$ for frame $f_{t}$. The action score $\alpha_{t}\left(f_{t}|k^{*}\right)$ and the action label $l_{t}$ for frame $f_{t}$ specifically are determined by

(4) $$\alpha_{t}\left(f_{t}|k^{*}\right)=\frac{\sum_{j\in \left\{j|f_{t}\in u_{j}\right\}}v_{j}^{spe}}{\left|\{j|j\in \left\{f_{t}\in u_{j}\right\}\}\right|}\;,$$

(5) $$l_{t}=\left\{\begin{array}{cc} k^{*}, & \;\mathrm{if}\;\alpha_{t}>\gamma ,\\ 0, & \;\mathrm{otherwise}, \end{array}\right.$$

where |{·}| denotes the cardinality of set {·}. We empirically set γ=0.6. $f_{s}\left(l_{t}\right)$ and $f_{e}\left(l_{t}\right)$ are assigned as the starting and ending frame of a series of consecutive frames sharing the same label $l_{t}$, respectively.

### 3.2. Spatial Action Segmentation

With the obtained temporal localization results, we further conduct spatial action segmentation. This problem is cast into a spatio-temporal energy minimization framework,

(6) $$E\left(B\right)=\sum_{s_{t,i}\in V}D_{i}\left(b_{t,i}\right)+\sum_{s_{t,i},s_{t,n}\in N_{i}}S_{in}^{intra}\left(b_{t,i},b_{t,n}\right)+\sum_{s_{t,i},s_{m,n}\in {\bar{N}}_{i}}S_{in}^{inter}\left(b_{t,i},b_{m,n}\right),$$

where $s_{t,i}$ is the *i*th superpixel in frame $f_{t}$. $D_{i}\left(b_{t,i}\right)$ composes the data term, denoting the cost of labeling $s_{t,i}$ with the label $b_{t,i}$ from a color and location based appearance model. $S_{in}^{intra}\left(b_{t,i},b_{t,n}\right)$ and $S_{in}^{inter}\left(b_{t,i},b_{m,n}\right)$ compose the smoothness term, constraining the segmentation labels to be spatially coherence from a color based intra-frame consistency model, and temporally consistent from a color based inter-frame consistency model, respectively. $N_{i}$ is the spatial neighborhood of $s_{t,i}$ in frame $f_{t}$. ${\bar{N}}_{i}$ is the temporal neighborhood of $s_{t,i}$ in adjacent frames $f_{t-1}$ and $f_{t+1}$. We compute the superpixels by using SLIC [55], due to its superiority in terms of adherence to boundaries, as well as computational and memory efficiency. However, the proposed method is not tied to any specific superpixel method, and one can choose others.

**Data Term.** The data term $D_{i}\left(b_{t,i}\right)$ defines the cost of assigning superpixel $s_{t,i}$ with label $b_{t,i}$ from an appearance model, which learns the color and location distributions of the action object and the backgrounds of video *V*. With a segmentation *B* for *V*, we estimate two color Gaussian Mixture Models (GMMs) and two location GMMs for the foregrounds and the backgrounds of *V*, respectively. The corresponding data term $D_{i}\left(b_{t,i}\right)$ based on color and location GMMs in Equation (6) is defined as

(7) $$D_{i}\left(b_{t,i}\right)=-\log\left(\beta h_{b_{t,i}}^{col}\left(s_{t,i}\right)+\left(1-\beta \right)h_{b_{t,i}}^{loc}\left(s_{t,i}\right)\right),$$

where $h_{b_{t,i}}^{col}$ denotes the two color GMMs, i.e., $h_{1}^{col}$ for the action object and $h_{0}^{col}$ for the background across video *V*. Similarly, $h_{b_{t,i}}^{loc}$ denotes the two location GMMs for the action object and the background across *V*, i.e., $h_{1}^{loc}$ and $h_{0}^{loc}$. β is a parameter controlling the contributions of color $h_{b_{t,i}}^{col}$ and location $h_{b_{t,i}}^{loc}$.

**Smoothness Term.** The action segmentation labeling *B* should be spatially consistent in each frame, and meanwhile temporally consistent throughout video *V*. Thus, we define the smoothness term by assembling an intra-frame consistency model and an inter-frame consistency model.

The intra-frame consistency model enforces the spatially adjacent superpixels in the same action frame to have the same label. Due to the fact that the adjacent superpixels either have similar color or distinct color contrast [56], the well-known standard contrast-dependent function [56,57] is exploited to encourage the spatially adjacent superpixels with similar color to be assigned with the same label. Then, $S_{iu}^{intra}\left(b_{t,i},b_{t,n}\right)$ in Equation (6) is defined as

(8) $$S_{in}^{intra}\left(b_{t,i},b_{t,n}\right)={𝟙}_{\left[b_{t,i}\neq b_{t,n}\right]}\exp(-||c_{t,i}-c_{t,n}{\left|\right|}_{2}^{2}),$$

where the characteristic function ${𝟙}_{\left[b_{t,i}\neq b_{t,n}\right]}=1$ when $b_{t,i}\neq b_{t,n}$, and 0 otherwise. $b_{t,i}$ and $b_{t,n}$ are the segmentation labels of superpixels $s_{t,i}$ and $s_{t,n}$, respectively. c is the color vector of the superpixel.

The inter-frame consistency model encourages the temporally adjacent superpixels in consecutive action frames to have the same label. As the temporally adjacent superpixels should have similar color and motion, we use the Euclidean distance between the motion distributions of temporally adjacent superpixels along with the above contrast-dependent function in Equation (8) to constrain the labels of them to be consistent. In Equation (6), $S_{in}^{inter}\left(b_{t,i},b_{m,n}\right)$ is then defined as

(9) $$S_{in}^{inter}\left(b_{t,i},b_{m,n}\right)={𝟙}_{\left[b_{t,i}\neq b_{m,n}\right]}(\exp(-\left|\right|c_{t,i}-c_{m,n}{\left|\right|}_{2}^{2})+\exp(-||h_{t,i}^{m}-h_{m,n}^{m}{\left|\right|}_{2})),$$

where $h^{m}$ is the histogram of oriented optical flow (HOOF) [58] of the superpixel.

**Optimization.** With $D_{i}\left(b_{t,i}\right)$, $S_{in}^{intra}\left(b_{t,i},b_{t,n}\right)$ and $S_{in}^{inter}\left(b_{t,i},b_{m,n}\right)$, we leverage graph cut [24] to minimize the energy function in Equation (6), and can obtain a new segmentation *B* for video *V*.

### 3.3. Iterative and Alternating Optimization

With an initial spatial segmentation $B_{o}$ of video *V* using saliency [23], the temporal action localization first pinpoints the starting frame $f_{s}\left(k\right)$ and the ending frame $f_{s}e\left(k\right)$ of a target action *k* from an untrimmed video *V* by a coarse-to-fine action localization strategy, and then the spatial action segmentation further produces the spatial per-frame segmentation *B* by focusing on the action frames identified by the temporal action localization. With the new segmentation *B* of video *V*, the overall optimization alternates between the temporal action localization and spatial action segmentation. Upon the practical convergence of this iterative process, the final results *B* are obtained. Naturally, the temporal action localization and spatial action segmentation benefit each other. In the experiments, we terminate the iterative optimization after practical convergence is observed, i.e., the relative variation between two successive spatio-temporal action localization results are smaller than 0.001.

## 4. Experiments and Discussion

### 4.1. Datasets and Evaluation Protocol

We conduct extensive experiments on multiple datasets to evaluate the efficacy of the proposed spatio-temporal action localization detector Segment-tube, including (1) temporal action localization task on the THUMOS 2014 dataset [25]; (2) spatial action segmentation on the SegTrack dataset [26,27]; and (3) spatio-temporal action localization task on the newly proposed ActSeg dataset.

The average precision (AP) and mean average precision (mAP) are employed to evaluate the temporal action localization performance. If an action is assigned the same category label with the ground truth, and, simultaneously, its predicted temporal range overlaps the ground truth at a ratio above a predefined threshold (e.g., 0.5). Such temporal localization of an action is deemed correct.

The intersection-over-union (IoU) value is utilized to evaluate the spatial action segmentation performance, and it is defined as

(10) $$\mathrm{IoU}=\frac{\left|Seg\cap GT\right|}{\left|Seg\cup GT\right|},$$

where Seg denotes the binary segmentation result obtained by a detector, GT denotes the binary ground truth segmentation mask, and |·| denotes the cardinality ( i.e., pixel count).

### 4.2. Implementation Details

**Training the proposal network**. The proposal network is to predict each video clip $u_{j}$ either contains an action ($l_{j}^{fla}=1$) or the background ($l_{j}^{fla}=0$), and thus can remove the background video clips, as described in Section 3.1. We build the training data as follows to train the proposal network. For each video clip from trimmed videos, we assign its action label as 1, denoting it contains some action *k* ($\forall k\in \left\{1,\ldots ,K\right\}$). For each video clip from untrimmed videos with temporal annotations, we set its label by using the IoU value between it and the ground truth action instances. If the IoU value is higher than 0.75, we assign the label as 1, denoting that it contains an action; if the IoU value is lower than 0.25, we assign the label as 0, denoting that it does not contain an action.

The 3D ConvNets [4] components (as shown in Figure 2) are pre-trained on the training split of the Sports-1M dataset [59], and used as the initializations of our proposal and classification networks. The output of the softmax layer in the proposal network is of two dimensions, which corresponds to either an action or the background. In all the following experiments, the batch size is fixed at 40 during the training phase, and the initial learning rate is set at $10^{-4}$ with a learning rate decay of factor 10 every 10 K iterations.

For the LSTM component, the activation feature of the fully-connected 7th layer of the 3D ConvNets [4] is used as the input to the LSTM. The learning batch size is set to be 32, where each sample in the minibatch is a sequence of ten 16-frame video clips. We use RMSprop [60] with a learning rate of $10^{-4}$, a momentum of 0.9 and a weight decay factor of $5\times 10^{-4}$. The number of iterations depends on the size of the dataset, and will be elaborated in the following temporal action localization experiments.

**Training the classification network**. The classification network is to further predict whether each video clip $u_{j}$ contains a specific action ($l_{j}^{spe}\in {\left\{k\right\}}_{k=0}^{K}$) or not, as described in Section 3.1. The training data for the classification network is built similarly to that of the proposal network. The only difference is that, for the saliency-aware positive video clip, we assign its label as a specific action category $k\in \left\{1,\ldots ,K\right\}$ (e.g., “LongJump”), instead of 1 for training the above proposal network.

As to the 3D ConvNets [4] (see Figure 2), we train a classification model with *K* actions plus one additional “background” category. The learning batch size is fixed at 40, the initial learning rate is $10^{-4}$ and the learning rate is divided by 2 after every 10 K iterations.

To train the LSTM, the activation feature of the fully-connected 7th layer of the 3D ConvNets [4] is fed to the LSTM. We fix the learning batch size at 32, where each sample in the minibatch is a sequence of ten 16-frame video clips. We also use RMSprop [60] with a learning rate of $10^{-4}$, a momentum of 0.9 and a weight decay factor of $5\times 10^{-4}$.

### 4.3. Temporal Action Localization on the THUMOS 2014 Dataset

We first evaluate the temporal action localization performance of the proposed Segment-tube detector on the THUMOS 2014 dataset [25], which is dedicated to localizing actions in long untrimmed videos involving 20 actions. The training set contains 2755 trimmed videos and 1010 untrimmed validation videos. For the 3D ConvNets training, the fine-tuning stops at 30 k for the two networks. For the LSTM training, the number of training iterations is 20 k for two networks. For testing, we use 213 untrimmed videos that contain relevant action instances.

Five existing temporal action localization methods, i.e., AMA [1], FTAP [9], ASLM [10], SCNN [11], and ASMS [15], are included as competing algorithms. AMA [1] combines iDT features and frame-level CNN features to train a SVM classifier. FTAP [9] leverages high recall temporal action proposals. ASLM [10] uses a length and language model based on traditional motion features. SCNN [11] is an end-to-end segment-based 3D ConvNets framework, including proposal, classification and localization network. ASMS [15] localizes actions by searching for the structured maximal sum.

**Figure 4:**
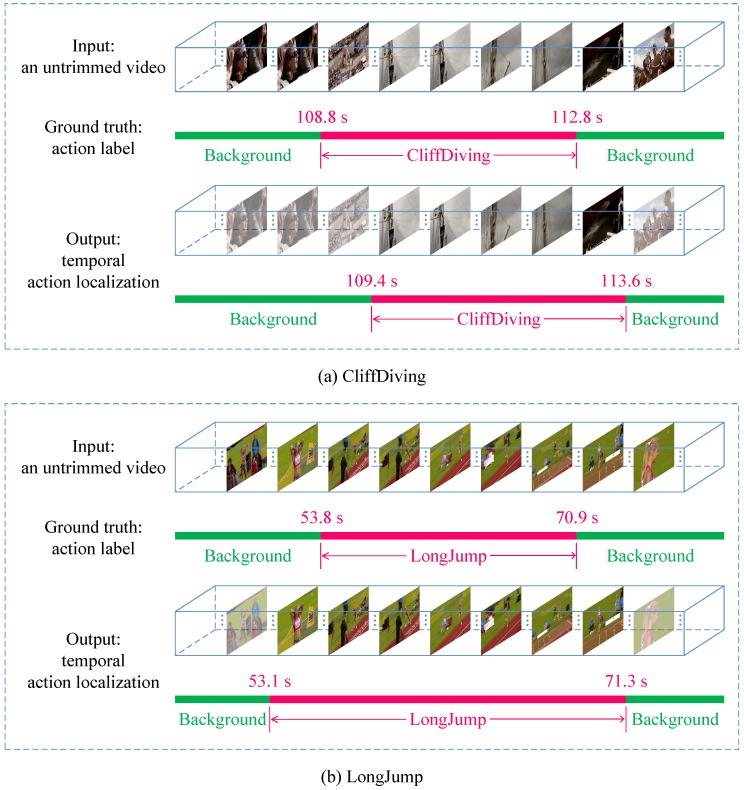
Qualitative temporal action localization results of the proposed Segment-tube detector for two action instances, i.e., (**a**) CliffDiving and (**b**) LongJump, in the testing split of the THUMOS 2014 dataset, with intersection-over-union (IoU) threshold being 0.5.

The mAP comparisons are summarized in Table 1, which demonstrate that the proposed Segment-tube detector evidently outperforms the five competing algorithms with IoU being 0.3 and 0.5, and is marginally inferior to SCNN [11] with IoU threshold being 0.4. We also present the qualitative temporal action localization results of the proposed Segment-tube detector for two action instances of the testing split from the THUMOS 2014 dataset in Figure 4, with IoU threshold being 0.5.

### 4.4. Spatial Action Segmentation on the SegTrack Dataset

We then evaluate the performance of spatial action segmentation from trimmed videos on the SegTrack dataset [26,27]. The dataset contains 14 video sequences with lengths varying from 21 to 279 frames. Every frame is annotated with a pixel-wise ground-truth segmentation mask. Due to the limitation of the competing methods [47,48,52], a subset of eight videos are selected, all of which contains only one action object.

We compare our proposed Segment-tube detector with three state-of-the-art video object segmentation methods, i.e., VOS [48], FOS [47] and BVS [52]. VOS [48] automatically discovers and groups key segments to isolate the foreground object. FOS [47] separates the foreground object based on an efficient initial foreground estimation and a foreground-background labeling refinement. BVS [52] obtains the foreground object via bilateral space operations.

**Figure 5:**
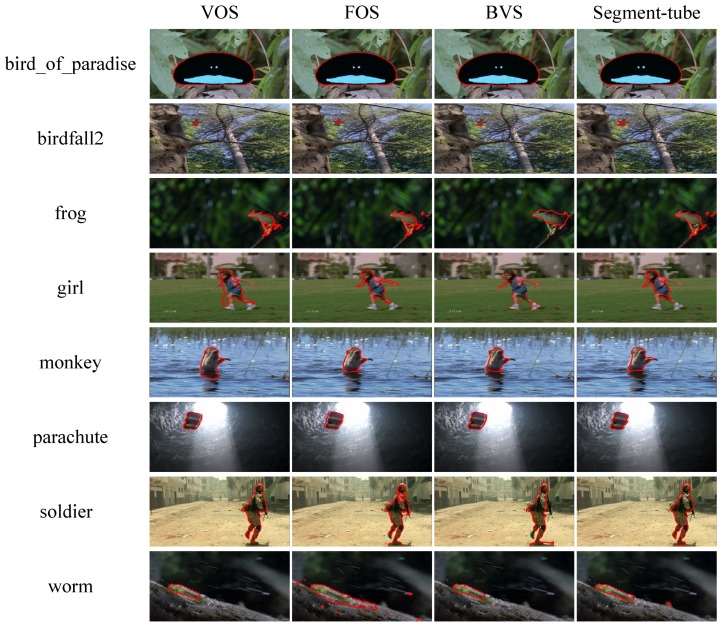
Example results of three state-of-the-art video object segmentation methods (VOS [48], FOS [47] and BVS [52]) and our proposed Segment-tube detector on the SegTrack dataset [26,27].

The IoU value comparison of VOS [48], FOS [47], BVS [52] and our proposed Segment-tube detector on the SegTrack dataset [26,27] is presented in Table 2. Some example results of them are given in Figure 5, where the predicted segmentation masks are visualized by polygons with red edges. As is shown in Table 2, our method significantly outperforms VOS [47] and FOS [47], and performs better than BVS [52] with a small margin of 2.3. The performance of BVS [52] could possibly due to its exploitation of the first-frame segmentation mask to facilitate the subsequent segmentation procedure.

### 4.5. Spatio-Temporal Action Localization on the ActSeg Dataset

**ActSeg dataset.** To fully evaluate the proposed spatio-temporal human action localization detector and to build a benchmark for future research, a new ActSeg dataset is introduced in this paper, including both untrimmed and trimmed videos. The list of action classes are presented in Table 3, including single person actions (e.g., “ArabequeSpin”, “PoleVault”, “NoHandWindmill”) and multi-person actions (e.g., “DeathSpirals”).

All raw videos are downloaded from YouTube. Typical untrimmed videos contain approximately 10–120 s of irrelevant frames prior and/or after the specific action. The trimmed videos are pruned so that they only contain relevant action frames. We have recruited 30 undergraduate students to independently decide whether a specific action is present (positive label) in the original video or not (negative label). If four or more positive labels are recorded, the original video is accepted in the ActSeg dataset and the time boundaries of the action are determined as follows. Each accepted video is independently distributed to 3~4 undergraduate students for manual annotation (for both the temporal boundaries and per-frame pixel-wise segmentation labels) and an additional quality comparison is carried out for each accepted video by a graduate student and the best annotation is selected as the ground truth.

**Figure 6:**
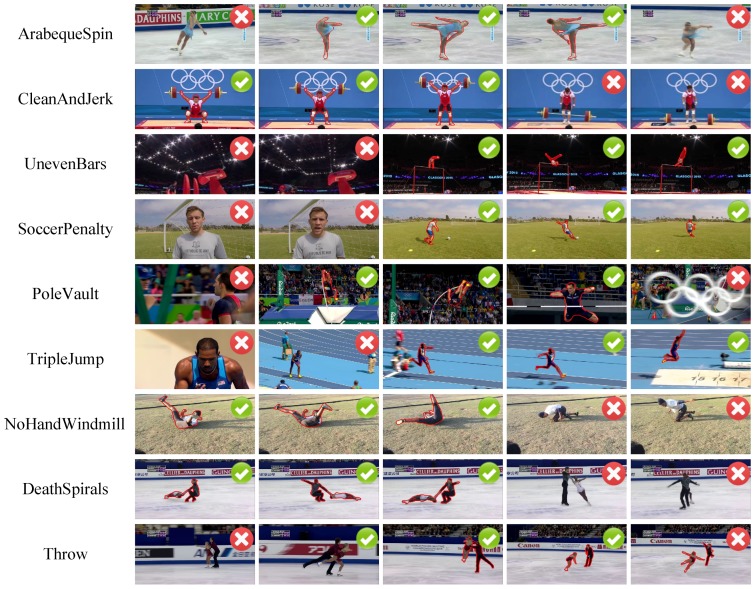
Sample frames and their ground truth annotations in the ActSeg dataset. Action frames are marked by green check marks and the corresponding boundaries are marked by polygons with red edges. The background (irrelevant) frames are marked by red cross marks.

The complete ActSeg dataset contains 641 videos in nine human action categories. There are 446 untrimmed videos and 110 trimmed videos in its training split, 85 untrimmed videos and no trimmed video in its testing split. Table 3 presents detailed statistics for the untrimmed/trimmed video distribution in each category. Some typical samples with their corresponding ground truth annotations are illustrated in Figure 6.

**Mixed Dataset.** To maximize the number of videos in each category (see Table 3), a mixed dataset is constructed by combining videos of identical action categories from multiple datasets. The training split of the mixed dataset consists of all 446 untrimmed videos and 110 trimmed videos in the proposed ActSeg dataset, 791 trimmed videos from the UCF-101 dataset [61], and 90 untrimmed videos from the THUMOS 2014 dataset [25]. The testing split of the mixed dataset consists of all the 85 untrimmed videos from the testing split of the proposed ActSeg dataset.

**Temporal Action Localization.** SCNN [11] and ARCN [8] are used as competing temporal action localization methods. All three methods are trained on the training split of the mixed dataset. For the 3D ConvNets, the fine-tuning stops at 20 k for the proposal and classification networks. For LSTM training, the number of training iterations is 10 k for the two networks. Table 4 presents the mAP comparisons of SCNN [11], ARCN [8] and our proposed Segment-tube detector on the testing split of the mixed dataset, with IoU threshold being 0.3, 0.4, and 0.5, respectively. The results show that our proposed Segment-tube method achieves the best mAP with all three IoU thresholds. These manifest the efficacy of the proposed coarse-to-fine action localization strategy and also the Segment-tube detector.

**Figure 7:**
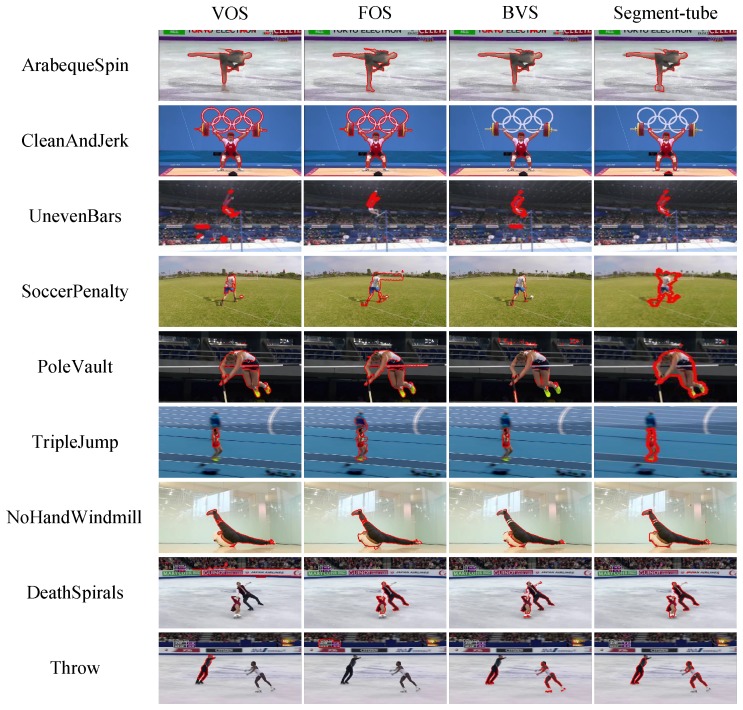
Example results of three video object segmentation methods (VOS [48], FOS [47] and BVS [52]) and our proposed Segment-tube detector on the ActSeg dataset.

**Spatial Action Segmentation.** The spatial action segmentation task is implemented entirely on the ActSeg dataset, with three competing video object segmentation methods, i.e., VOS [48], FOS [47] and BVS [52]. The IoU score comparisons of them are summarized in Table 5. Figure 7 presents some example results of them, where the predicted segmentation masks are visualized by polygons with red edges. Note that the IoU scores are computed only on frames that contain the target action, which are localized by the temporal action localization of the proposed Segment-tube detector.

The results in Table 5 demonstrate that the Segment-tube detector evidently outperforms VOS [48], FOS [47], and the label propagation based method BVS [52]. On the videos of PoleVault and TripleJump categories, the IoU scores of all the methods are low, which is mainly due to severe occlusions.

**Figure 8:**
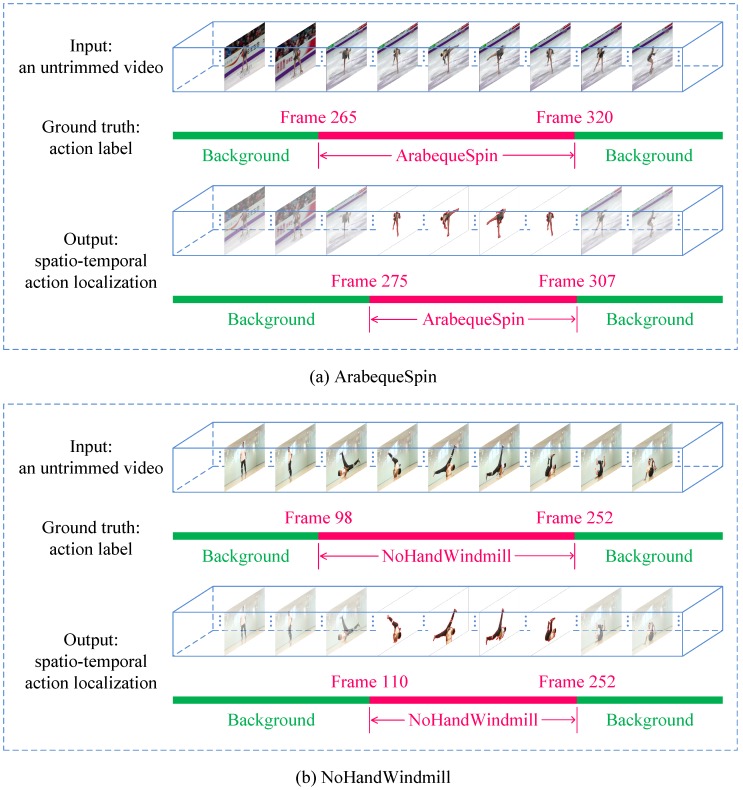
Qualitative spatio-temporal action localization results of the proposed Segment-tube for two action instances, i.e., (**a**) ArabequeSpin and (**b**) NoHandWindmill, in the testing split of the ActSeg dataset, with intersection-over-union (IoU) threshold being 0.5.

Because existing methods either implement temporal action localization or spatial action segmentation, but never achieve both of them simultaneously, we do not include performance comparisons of joint spatio-temporal action localization with per-frame segmentations. To supplement this, we further present the qualitative spatio-temporal action localization results of the proposed Segment-tube for two action instances in the ActSeg dataset (testing split) in Figure 8.

To summarize, the experimental results on the above three datasets reveal that the Segment-tube detector produces superior results to existing state-of-the-art methods, which verifies its ability of collaboratively and simultaneously implementing spatial action segmentation and temporal action localization with untrimmed videos.

### 4.6. Efficiency Analysis

The segment-tube detector is highly computational efficient, especially comparing with other approaches that fuse multiple features. Most video clips containing pure background are eliminated by the proposal network, thus the computational cost with the classification network is significantly reduced. On a NVIDIA (NVIDIA Corporation, Santa Clara, CA, USA) Tesla K80 GPU with 12 GB memory, the amortized time of processing one batch (approximately 40 sampled video clips) is approximately one second. Video clips have variable length and 16 frames are uniformly sampled from each video clip. Each input for the 3D ConvNets is a sampled video clip of dimension 3×16×171×128 (RGB channels × frames × width × height).

## 5. Conclusions

We propose the spatio-temporal action localization detector Segment-tube, which simultaneously localizes the temporal action boundaries and per-frame spatial segmentation masks in untrimmed videos. It overcomes the common limitation of previous methods that either implement only temporal action localization or just (spatial) video object segmentation. With the proposed alternating iterative optimization scheme, temporal localization and spatial segmentation could be achieved collaboratively and simultaneously. Upon practical convergence, a sequence of per-frame segmentation masks with precise starting/ending frames are obtained. Experiments on three datasets validate the efficacy of the proposed Segment-tube detector and manifest its ability to handle untrimmed videos.

The proposed method is currently dedicated to spatio-temporal localization of a single specific action in untrimmed videos, and we are planning to extend it to simultaneous spatio-temporal localization of multiple actions with per-frame segmentations in our future work. One potential direction is the generation of multiple action category labels in the classification network of the coarse action localization step, followed by independent fine action localization and spatial action segmentation for each action category.

## Figures and Tables

**Table 1:** Mean average precision (mAP) comparisons of five state-of-the-art temporal action localization methods and our proposed Segment-tube detector on the THUMOS 2014 dataset [25]. mAP values are in percentage. Higher values are better.

IoU Threshold	0.3	0.4	0.5
AMA [1]	14.6	12.1	8.5
FTAP [9]	-	-	13.5
ASLM [10]	20.0	23.2	15.2
SCNN [11]	36.3	28.7	19.0
ASMS [15]	36.5	27.8	17.8
Segment-tube	39.8	27.2	20.7

**Table 2:** Intersection-over-union (IoU) value comparison of three state-of-the-art video object segmentation methods (VOS [48], FOS [47] and BVS [52]) and our proposed Segment-tube detector on the SegTrack dataset [26,27]. IoU values are in percentage. Higher values are better.

Algorithm	VOS [48]	FOS [47]	BVS [52]	Segment-Tube
bird_of_paradise	92.4	81.8	91.7	93.1
birdfall2	49.4	17.5	63.5	66.7
frog	75.7	54.1	76.4	70.2
girl	64.2	54.9	79.1	81.3
monkey	82.6	65.0	85.9	86.9
parachute	94.6	76.3	93.8	90.4
soldier	60.8	39.8	56.4	64.5
worm	62.2	72.8	65.5	75.2
**Average**	72.7	57.8	76.5	78.8

**Table 3:** Statistics on total, untrimmed and trimmed videos in each category of the ActSeg dataset.

Number	Total Videos	Untrimmed Videos	Trimmed Videos
ArabequeSpin	68	58	10
CleanAndJerk	73	61	12
UnevenBars	67	57	10
SoccerPenalty	82	67	15
PoleVault	72	59	13
TripleJump	62	50	12
NoHandWindmill	68	57	11
DeathSpirals	78	66	12
Throw	71	56	15
**Sum**	641	531	110

**Table 4:** Mean average precision (mAP) comparisons of two temporal action localization methods (SCNN [11] and ARCN [8]) and our proposed Segment-tube detector on the testing split of the mixed dataset, with intersection-over-union (IoU) threshold being 0.3, 0.4, and 0.5, respectively. mAP values are in percentage. Higher values are better.

IoU Threshold	0.3	0.4	0.5
ARCN [8]	39.1	33.8	17.2
SCNN [11]	41.0	35.9	18.4
Segment-tube	42.6	37.5	21.2

**Table 5:** Intersection-over-union (IoU) value comparisons of three video object segmentation methods (VOS [48], FOS [47] and BVS [52]) and our proposed Segment-tube detector on the ActSeg dataset. IoU values are in percentage. Higher values are better.

Video	VOS [48]	FOS [47]	BVS [52]	Segment-Tube
ArabequeSpin	53.9	82.5	64.0	83.4
CleanAndJerk	20.1	50.0	85.9	87.8
UnevenBars	12.0	40.3	59.0	56.5
SoccerPenalty	54.4	38.5	59.8	54.7
PoleVault	38.9	41.2	42.6	49.8
TripleJump	30.6	36.1	33.5	58.4
NoHandWindmill	77.1	73.3	81.8	87.9
DeathSpirals	1	66.7	77.9	66.5
Throw	33.8	2	58.7	56.2
**Average**	35.8	47.8	62.6	66.8

## Abbreviations

The following abbreviations are used in this manuscript:

CNNs	Convolutional Neural Networks
ConvNets	Convolutional Neural Networks
I3D	Inflated 3D ConvNet
ResNet	Deep Residual Convolutional Neural Networks
LSTM	Long-Short Temporal Memory
RNNs	Recurrent Neural Networks
HOG	Histograms of Image Gradients
HOF	Histogram of Flow
iDT	improved Dense Trajectory
FV	Fisher Vector
VLAD	Vector of Linearly Aggregated Descriptors
FCNs	Fully Convolutional Networks
GMMs	Gaussian Mixture Models
HOOF	Histogram of Oriented Optical Flow
AP	Average Precision
mAP	mean Average Precision
IoU	Intersection-over-Union
RGB	Red Green Blue
